# The cell cycle-regulated cytoplasmic kinase, TgCRCK1, is required for efficient propagation of human protozoan pathogen, *Toxoplasma gondii*

**DOI:** 10.1128/spectrum.02691-25

**Published:** 2025-12-31

**Authors:** Dima Hajj Ali, Ramu Anandakrishnan, Frank O. Aylward, Rajshekhar Y. Gaji

**Affiliations:** 1 Department of Biomedical Sciences and Pathobiology, Virginia-Maryland College of Veterinary Medicine 70732, Blacksburg, Virginia, USA; 2 Department of Biomedical Sciences, Edward Via College of Osteopathic Medicine (VCOM) 41066, Blacksburg, Virginia, USA; 3 Department of Biological Sciences, Virginia Tech 1757, Blacksburg, Virginia, USA; University of Illinois Urbana-Champaign, Urbana, Illinois, USA

**Keywords:** *Toxoplasma gondii*, protein kinases, cell cycle regulation, lytic cycle, auxin-inducible degron system

## Abstract

*Toxoplasma gondii* is an obligate intracellular parasite that relies on a complex network of protein kinases to regulate essential processes such as invasion, replication, and egress. The *Toxoplasma* genome encodes approximately 159 kinases, yet only a small subset has been characterized. Our group is interested in defining the role of cell cycle-regulated kinases important for *Toxoplasma* fitness. In this study, we investigated the role of an uncharacterized, cell cycle-regulated cytoplasmic kinase, which we named TgCRCK1, in parasite biology and pathogenesis. Using endogenous tagging and immunofluorescence assays, we demonstrated that TgCRCK1 is a cytosolic kinase exhibiting a cell cycle-dependent expression pattern. Conditional depletion of TgCRCK1 via an auxin-inducible degron system impaired parasite growth, primarily due to defects in cell division and invasion. Transcriptomic analysis of TgCRCK1-deficient parasites revealed 93 differentially expressed genes, many involved in metabolism, gene expression, and intracellular transport. Although TgCRCK1 is essential for parasite growth, its depletion did not reduce *Toxoplasma* virulence in the mouse model. Collectively, these findings identify TgCRCK1 as a key factor contributing to *Toxoplasma* propagation.

**IMPORTANCE:**

*Toxoplasma gondii* is the primary causative agent of toxoplasmosis, infecting a broad range of warm-blooded animals, including humans. The parasite depends on a complex network of protein kinases for growth and pathogenesis, yet the functions of many remain uncharacterized. In this study, we present the initial characterization of TgCRCK1, a cytoplasmic kinase with temporally regulated expression. Our results demonstrate that loss of TgCRCK1 impairs parasite growth *in vitro* by disrupting parasite division and host-cell invasion. However, studies in an animal model indicate that TgCRCK1 is not essential for acute toxoplasmosis. Together, these findings provide foundational insights into the localization, expression, and function of TgCRCK1 in this important human pathogen.

## INTRODUCTION

*Toxoplasma gondii,* the causative agent of toxoplasmosis in humans, is a unicellular eukaryote classified within the phylum Apicomplexa (1, 2). This phylum includes several notable human and animal pathogens, such as *Plasmodium*, *Cryptosporidium*, *Babesia*, and *Sarcocystis* spp. (3). Cats act as the definitive hosts of *Toxoplasma* as the sexual phase of the life cycle of the parasite occurs in the intestinal epithelial cells of this species (4). The parasite is excreted in cat feces as oocysts, which act as the source of infection for intermediate hosts, primarily farm animals. Humans acquire *Toxoplasma* infection through multiple routes, including ingestion of contaminated meat from infected intermediate hosts, consumption of food or water contaminated with parasite oocysts, or via transplacental transmission from mother to fetus during pregnancy. In individuals with healthy immune systems, *Toxoplasma* infection is typically asymptomatic. However, the disease can be fatal in immunocompromised individuals, including cancer patients undergoing immunosuppressive therapy and HIV-positive persons. Furthermore, primary exposure to *Toxoplasma* during pregnancy can result in miscarriage or congenital abnormalities in the newborn (1, 2, 5–7). Currently, no vaccine exists to prevent *Toxoplasma* infection, and the drugs used to treat the acute form of the disease are associated with adverse side effects (8).

The pathology of toxoplasmosis primarily results from extensive tissue damage caused by the rapid and prolific multiplication of the parasite within infected individuals (8, 9). Specifically, *Toxoplasma*, an obligate intracellular pathogen, actively invades host cells and resides within a protective parasitophorous vacuole. The parasite then replicates by endodyogeny, a process in which two daughter parasites bud within the mother parasite (10–12). *Toxoplasma* continues to multiply until the host cell becomes filled with tachyzoites. Approximately 2 days post-infection, the newly formed daughter cells egress, leading to rupture of the infected host cell and subsequent invasion of neighboring cells. Because the infected host cell is destroyed during parasite egress, this intracellular replication process is commonly referred to as the lytic cycle (8, 9). Since the events of the lytic cycle—invasion, replication, and egress—are critical for the efficient propagation and survival of *Toxoplasma* within the host, identifying and characterizing parasite factors essential for *Toxoplasma* growth has become a priority for the development of novel therapeutics (8, 13, 14).

Kinases play critical roles in diverse cellular processes within eukaryotic cells, including regulation of gene transcription, translation, metabolism, cell division, motility, and responses to environmental signals. The *Toxoplasma* genome contains approximately 8,000 genes, of which 159 are predicted to encode kinases, representing about 2% of the genome (15). Members of this kinase family have been shown to be essential for key *Toxoplasma* processes, such as invasion, motility, cytoskeletal organization, endodyogeny, egress, and gene regulation (16–21). Although significant progress has been made in mining the *Toxoplasma* genome to understand the roles of kinases in parasite biology, the precise functions of many kinase family members remain largely undetermined.

In *Toxoplasma*, kinases have been found in different organelles, including the parasite nucleus, cytoplasm, plasma membrane, rhoptries, apicoplast, dense granules, pellicle, and the mitochondrion (15, 16, 20, 22–29). Although a large number of these kinases are constitutively expressed, some of these exhibit cell cycle regulation (20). A notable group of cell cycle-regulated kinases belongs to the Cdk-related kinase (CRK) family, which localizes to the parasite nucleus and plays a critical role in cell cycle progression (30). Other *Toxoplasma* kinases with periodic expression include TgMAPK1, TgMAPK2, TgARK1-3, TgNEK1, and TgTKL4; interestingly, many of these have been shown to be involved in parasite cell division (18, 31–36).

Our group aims to understand the role of cell cycle-regulated kinases that may be important for *Toxoplasma* growth and pathogenesis. In this study, we provide initial insights into the function of *Toxoplasma gondii* Cell-cycle Regulated Cytoplasmic Kinase 1 (TgCRCK1), a kinase predicted to be regulated during the cell cycle and important for parasite fitness (20). We show that the protein localizes to the parasite cytoplasm and is temporally regulated with exclusive expression in S and M/C phases. Phenotypic analysis of TgCRCK1 showed that its absence results in impaired parasite growth that is caused by defects in endodyogeny and reduced invasion competence. Furthermore, transcriptomic analysis suggested that loss of TgCRK1 results in dysregulation of gene expression profile in the parasite. However, despite its critical requirement for efficient parasite growth *in vitro*, TgCRCK1 depletion did not attenuate the parasite’s virulence in a murine infection model. Collectively, these findings provide novel insights into the role of this cell cycle-regulated kinase in *Toxoplasma* biology.

## RESULTS

### TGGT1_275610 is a cytoplasmic kinase expressed during the S and M/C phases of tachyzoite cell cycle

**Fig 1:**
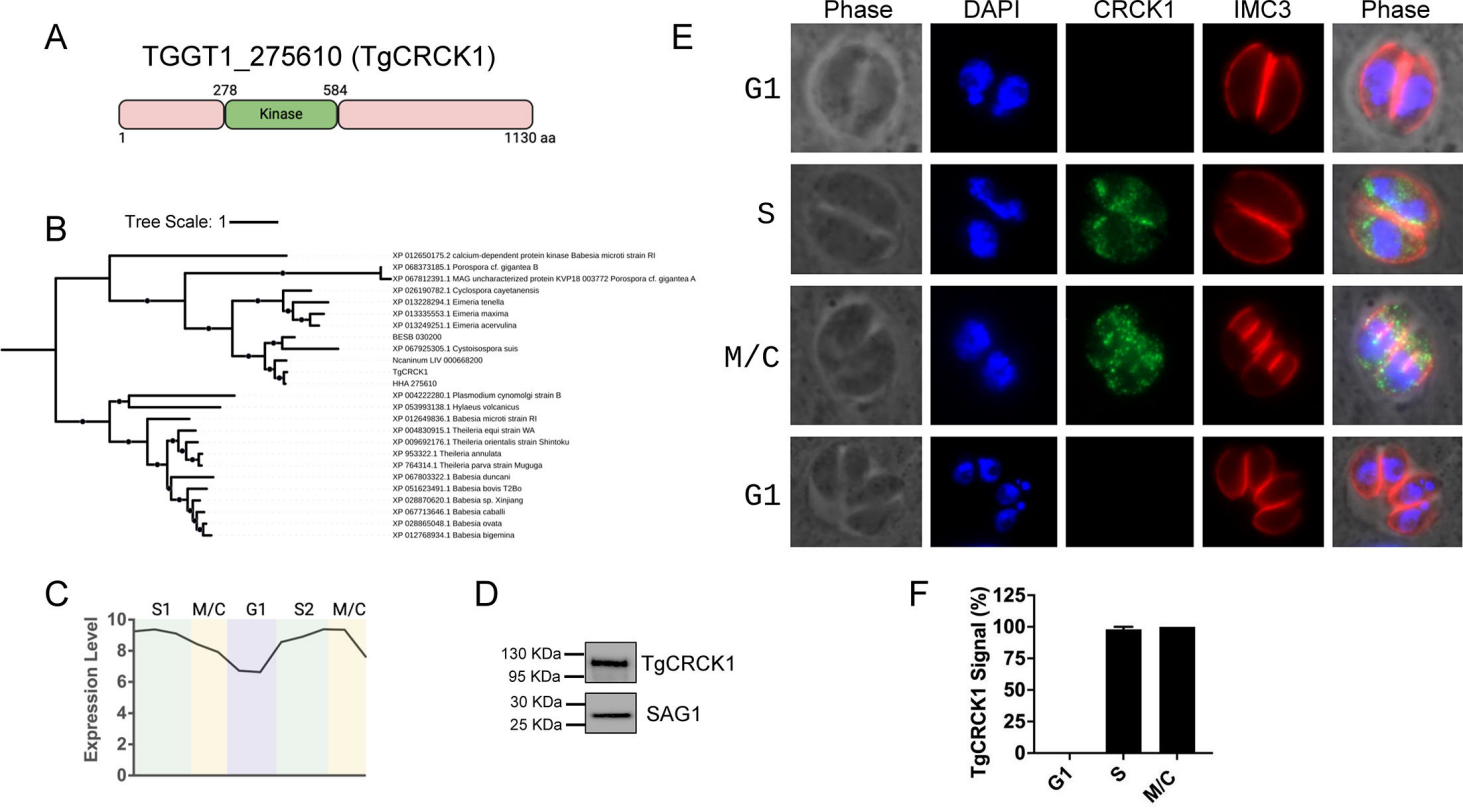
TgCRCK1 is a cell cycle-dependent cytoplasmic kinase. (**A**) Schematic representation of TgCRCK1 domain architecture, highlighting the relative position of the kinase domain. (**B**) Phylogenetic analysis of TgCRCK1 among related apicomplexan parasites. The alignments were generated using Muscle 5.1, and IQ-TREE v. 2.2.2.7 was used to produce the tree using the LG+F+R10 model (37). (**C**) Transcriptomic profile of TgCRCK1 across different cell cycle stages (39). (**D**) Western blot analysis of TgCRCK1.HA strain using an anti-HA antibody. TgSAG1 is used as a loading control. (**E**) Immunofluorescence analysis showing the localization of TgCRCK1 in intracellular parasites at various cell cycle stages using anti-HA antibody. TgIMC3 (red), a marker for the pellicle, was used to identify different cell cycle stages. DAPI was used to stain the nuclei. Scale bar, 2 μm. (**F**) Quantification of parasitophorous vacuoles in different cell cycle stages that showed TgCRCK1 signal. Data are presented as mean ± standard deviations from three independent experiments.

TGGT1_275610 is a *Toxoplasma* gene encoding a 1,130-amino acid protein with a kinase domain located in the distal region of the proximal half of the protein (Fig. 1A). Phylogenetic analysis (37) indicated that this kinase is highly conserved among members of the *Eimeriidae* family of apicomplexan parasites (*Hammondia hammondi, Besnoitia besnoiti, Cystoisospora suis,* and *Neospora caninum*) and exhibits only weak homology to piroplasmids, such as *Plasmodium, Babesia,* and *Theileria* (38). Notably, within the *Eimeriidae*, the kinase shows the greatest orthology with *Hammondia* species (Fig. 1B).

A previous transcriptomic study suggested that TGGT1_275610 is a cell cycle-regulated kinase, with peak expression during the S and M/C phases (40) (Fig. 1C). To determine its localization, we endogenously tagged TGGT1_275610 at the C-terminus with a hemagglutinin (HA) epitope using CRISPR/Cas9 technology (41). Western blot analysis with an anti-HA antibody detected a single band of the expected size (~118 kDa) in the tagged clone (Fig. 1D).

Next, we wanted to determine the localization of this kinase in the parasite, and hence, we performed immunofluorescence analysis of intracellular parasites. The results revealed that TgGT1_275610 localizes to parasite cytoplasm. Interestingly, the protein was not observed during the G1 stage of the parasite cell cycle. However, the protein makes an entrance in the S phase of the cell cycle, persists during M/C stages, and once parasites return to G1 phase, the protein again disappears (Fig. 1E). This stage-specific pattern was further supported by quantification of vacuoles exhibiting a TgTCRCK1 signal (Fig. 1F). We also observed that during the M/C phases, some fraction of the protein showed co-localization with the inner membrane complex (IMC) regions of the newly forming daughter cells (Fig. 1D).

Altogether, these results suggest that TGGT1_275610 is indeed a temporally regulated cytoplasmic kinase and its expression is confined to the S and M/C phases during tachyzoite cell cycle. Since this is a developmentally regulated kinase found in the parasite cytoplasm, we named this protein TgCRCK1.

### Generation of conditional knockdown strain of TgCRCK1

Next, we wanted to determine the role of TgCRCK1 in *Toxoplasma* propagation. Since a previous study that conducted genome-wide analysis to identify genes important for *Toxoplasma* fitness suggested that TgCRCK1 is an essential gene with a mean phenotype score of −4.25 (40), we wanted to generate a conditional knockdown mutant of this gene. Toward this goal, we used the recently developed auxin-inducible degron (AID) system (42). This system involves tagging the C-terminus of TgCRCK1 with a mini-AID (mAID) domain followed by an HA epitope in a parasite line stably expressing the auxin receptor TIR1 (42). In the absence of auxin, the mAID-tagged TgCRCK1 functions normally; however, upon auxin addition, the mAID tag targets the protein for ubiquitination and subsequent proteasomal degradation.

**Fig 2:**
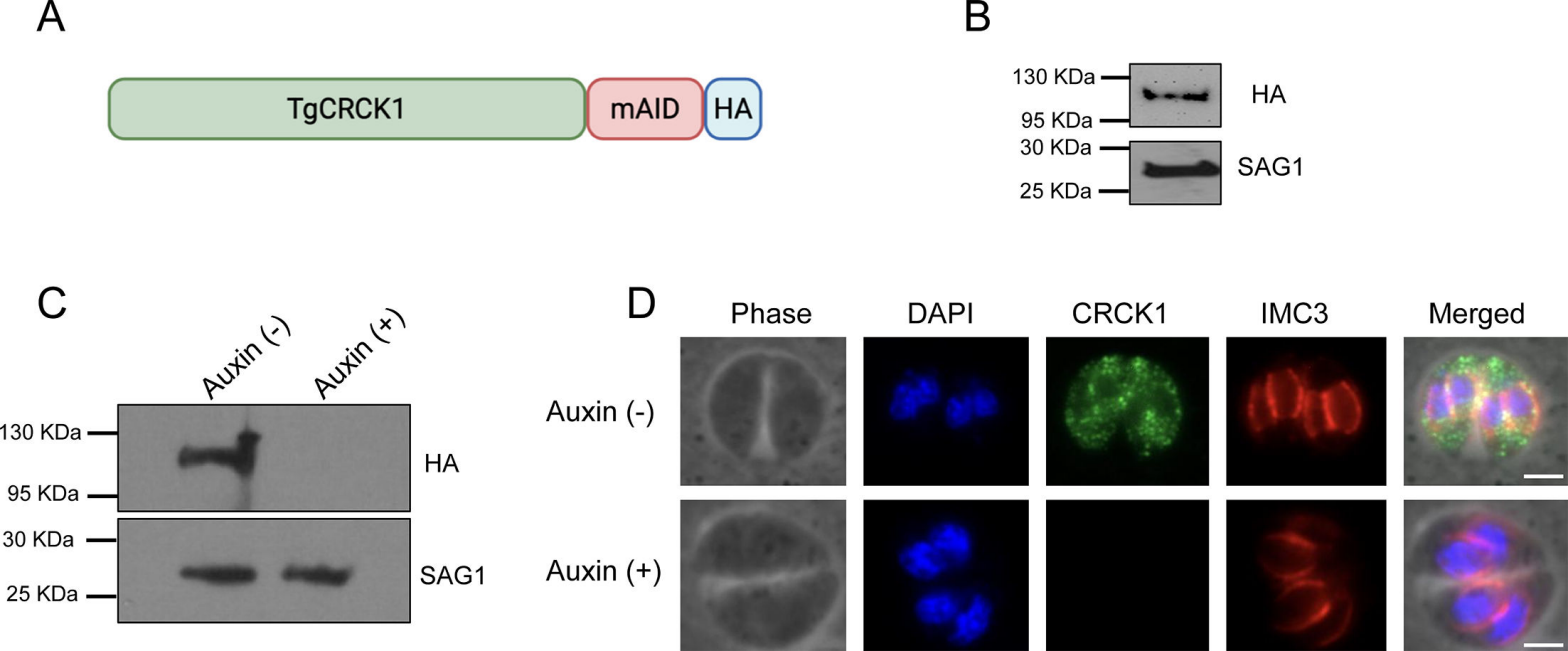
Establishment of TgCRCK1 conditional knockdown strain using the AID system. (**A**) Schematic representation of TgCRCK1 with the mAID-HA tag at the C terminus. (**B**) Immunoblot analysis of TgCRCK1.mAID.HA strain using anti-HA antibody. TgSAG1 is used as the loading control. (**C**) Western blot analysis of intracellular TgCRCK1.mAID.HA strain (treated with either vehicle control or auxin for 2 h) using an anti-HA antibody. TgSAG1 serves as the loading control. (**D**) Immunofluorescence analysis of TgCRCK1.mAID.HA parasites (treated with either vehicle control or auxin for 2 h) using anti-HA antibody. TgIMC3 used as an IMC marker. Scale bar, 2 μm.

Accordingly, we introduced the mAID-HA tag at the C terminus of TgCRCK1 by CRISPR-Cas9 technology to generate the TgCRCK1.mAID.HA strain as described in the Materials and Methods (Fig. 2A). Immunoblotting using anti-HA antibody revealed a single band of expected size for TgCRCK1 protein (Fig. 2B). Immunofluorescence analysis (IFA) confirmed that TgCRCK1.mAID.HA localizes to the parasite cytoplasm, consistent with our previous observation using the HA tag alone (Fig. 2D, top panel). To assess downregulation, parasites were treated with vehicle control or auxin, followed by immunoblotting. The results showed that TgCRCK1 completely disappears as early as 2 h in the presence of auxin (Fig. 2C). We also performed IFA analysis of TgCRCK1.mAID.HA strain using anti-HA antibody. We observed complete loss of TgCRCK1 protein in the parasites treated with auxin (Fig. 2D, bottom panel), thus suggesting successful establishment of conditional knockdown strain of TgCRCK1 protein.

### TgCRCK1 is important for parasite growth *in vitro*

**Fig 3:**
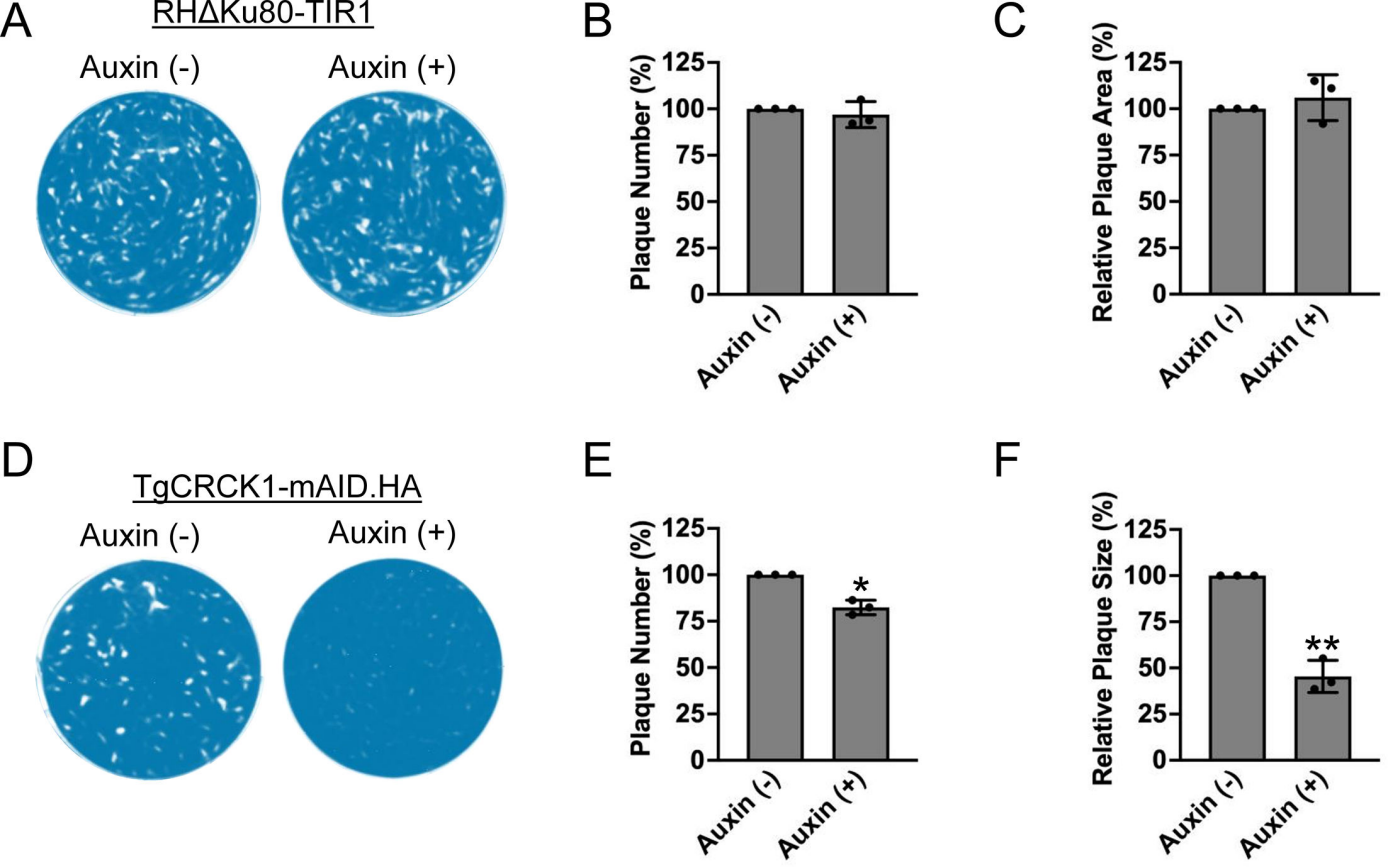
TgCRCK1 is critical for parasite growth *in vitro*. (**A**) Plaque assays were performed to examine the growth of the parental strain (RHΔku80-TIR1) in the presence or absence of auxin. Plaques are visible as clear zones on the background of crystal violet-stained HFF monolayer. Quantification of plaque numbers (**B**) and plaque size (**C**) for parental strain grown in the presence and absence of auxin. Data are presented as mean ± standard deviations from three independent experiments. ns, not significant. Unpaired two-tailed *t*-test. (**D**) Plaque assay showing the growth of TgCRCK1.mAID.HA strain cultured in presence or absence of auxin. Plaques are visible as clear zones on the background of a crystal violet-stained HFF monolayer. Quantification of plaque numbers (**E**) and plaque size (**F**) for TgCRCK1.mAID.HA parasites grown in the presence and absence of auxin. Data are presented as mean ± standard deviations from three independent experiments. *, *P* < 0.05 unpaired two-tailed *t*-test. **, *P* < 0.01 unpaired two tailed *t*-test.

To assess the impact of TgCRCK1 depletion on parasite replication and lytic cycle progression, we performed plaque assays on confluent human foreskin fibroblast (HFF) monolayers (Fig. 3A). Since the downregulation of TgCRCK1 involves culturing parasites in the presence of auxin, we first wanted to test if auxin treatment has any adverse effect on *Toxoplasma* growth. Towards this goal, we performed plaque assays for the parental strain (RHΔKu80-TIR1) in the presence or absence of auxin. The results showed that there was no significant difference in the number and size of the plaques formed by parental strain in the presence or absence of auxin (Fig. 3A through C). These findings suggested that auxin treatment does not affect *Toxoplasma* propagation *in vitro*. Next, to determine the contribution of TgCRCK1 to parasite growth, we performed plaque assays using the TgCRCK1.mAID.HA strain in the presence or absence of auxin. Importantly, we observed a significant reduction in the number (~18%) as well as the size (~55%) of the plaques with parasites lacking TgCRCK1 compared with the untreated parasites (Fig. 3D, E and F). These findings suggest that TgCRCK1 protein is indeed critical for efficient *Toxoplasma* growth *in vitro*.

### TgCRCK1-deficient parasites show a defect in host-cell invasion and parasite division

**Fig 4:**
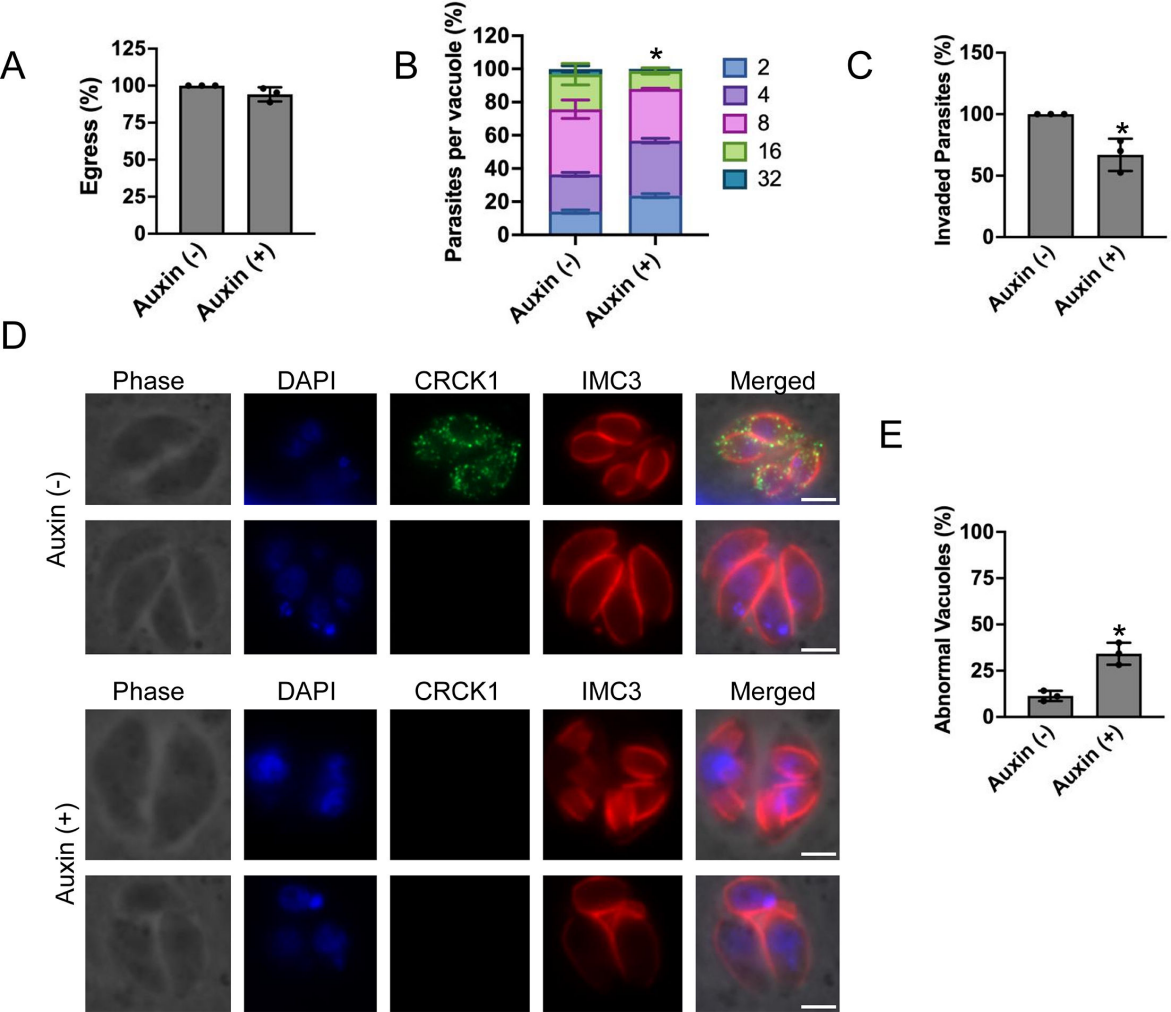
TgCRCK1-deficient parasites exhibit defects in host cell invasion and endodyogeny. TgCRCK1 parasites were subjected to lytic cycle assays, including induced egress (**A**), replication (**B**), and host-cell invasion (**C**) assays in the presence or absence of auxin. Data represent the mean and standard deviation results from three independent experiments, each performed with technical triplicates. ns, not significant. *, *P* < 0.05 Unpaired two-tailed *t* test. (**D**) Immunofluorescence examination of intracellular TgCRCK1.mAID.HA parasites treated with vehicle control (top two panels), or auxin (bottom two panels) followed by staining with anti-HA antibody and IMC3. scale bar, 2 μm. (**E**) Quantification of abnormal vacuoles in intracellular parasites treated with either vehicle control or auxin. Data are presented as mean ± standard deviations from three independent experiments. *, *P* < 0.05, Unpaired two-tailed *t*-test.

An impairment of plaque formation can be caused by defects in one or more steps of the parasite lytic cycle, including host-cell invasion, egress, or cell division. Therefore, we next sought to determine which aspect of the lytic cycle was impaired in parasites lacking TgCRCK1. We first performed egress assays using calcium ionophore A23187. However, we did not see any significant difference in egress capability of parasites treated either with vehicle control or auxin, thus indicating that TgCRCK1 does not appear to play any role in this event (Fig. 4A). We next assessed parasite replication using standard doubling assays (23), and we observed that 24 h post-infection, auxin treatment resulted in fewer numbers of parasites per vacuole compared with wild-type parasites (Fig. 4B). These results suggest that TgCRCK1 is required for efficient intracellular growth of *Toxoplasma*. Further, we also performed parasite invasion assay into host cells, and we found there is moderate reduction (~33%) in the number of invaded parasites with auxin-treated parasites compared with the control (Fig. 4C). These results suggest that TgCRCK1 is important for the *Toxoplasma* host-cell invasion process.

We also examined cell division in parasites treated with auxin or the vehicle control. We noticed that untreated parasites were able to undergo endodyogeny leading to the formation of normal daughter cells as expected (Fig. 4D). However, in parasites treated with auxin, we observed a significant increase in the number of vacuoles that showed abnormal features (Fig. 4D and E). This included multiple buddings in a single mother parasite as well as an odd number of parasites per vacuole (Fig. 4D and E). Additionally, TgCRCK1-deficient parasites showed defects in centrosome division, characterized by discrepancies between the number of centrosomes and parasite nuclei (Fig. S1A). We also observed abnormal apical cap formation in daughter cells lacking TgCRCK1, which may contribute to the irregular parasite numbers per vacuole (Fig. S1B). Collectively, these findings indicate that TgCRCK1 plays a critical role in *Toxoplasma* endodyogeny.

### Depletion of TgCRCK1 alters global gene expression in *T. gondii*

**Fig 5:**
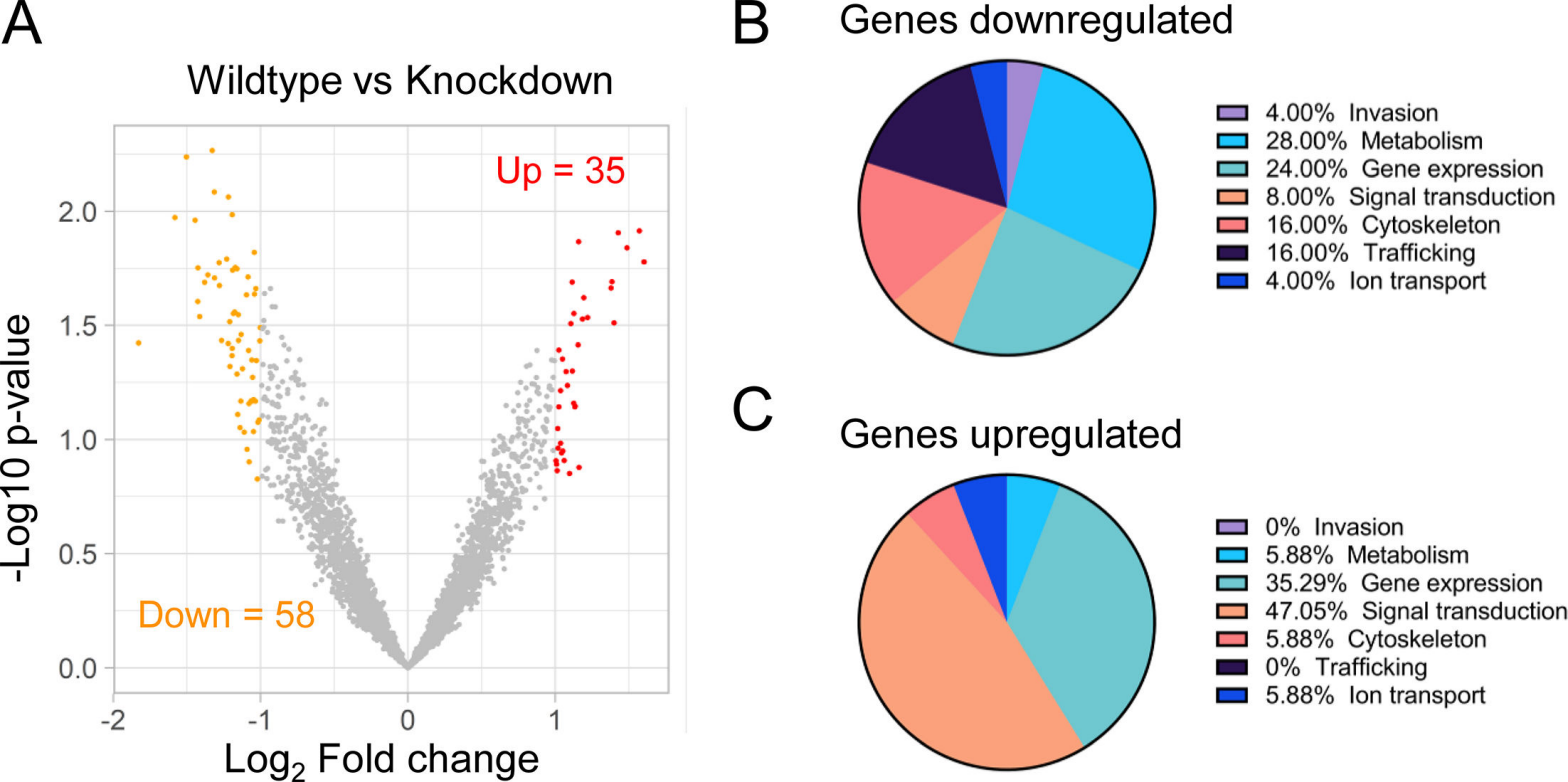
Loss of TGGT1_275610 results in dysregulation of gene expression in *Toxoplasma*. (**A**) Volcano plot illustrating the statistical significance (−log_10_ *P*-value) versus fold change (log_2_) in the gene expression of intracellular parasites treated with vehicle control or auxin. Differentially expressed genes (log_2_ fold change ≥ 2) are shown, with downregulated genes (*n* = 58) indicated in red on the left and upregulated genes (*n* = 35) in red on the right. (**B**) and (**C**) Pie charts showing the functional classification of downregulated (**B**) and upregulated (**C**) genes. Gene classifications were based on known or putative functions, derived from conserved domains.

To determine whether loss of TgCRCK1 affects gene expression in *Toxoplasma*, RNA sequencing (RNA-seq) analysis was performed on intracellular parasites. We purified RNA from TgCRCK1.mAID-HA parasites treated with vehicle control or auxin and performed transcriptomic analysis to assess the effects of loss of TgCRCK1 on gene expression globally. The RNA sequencing studies revealed that there were a total of 93 genes that were differentially expressed in parasites lacking TgCRCK1. Of these, 58 genes were downregulated, while 35 genes were upregulated (Fig. 5A and Table S2). To gain functional insights, we manually categorized the differentially expressed genes according to their known or predicted functions and also their cell cycle expression profile (43, 44). Among the regulated genes, a large percentage were related to parasite metabolism, while the upregulated gene set contained a major percentage of genes involved in gene expression (Fig. 5B and C and Table S2). Within the downregulated data set, 32 genes are constitutively expressed, 13 genes show cell cycle regulation, and there are no data available for the remaining 13 genes. Among the upregulated genes, 24 genes show constitutive expression, 10 genes exhibit temporal expression profile, and no data are available for one gene (Table S2). Together, these findings indicate that loss of TgCRCK1 does cause transcriptional changes in the parasite.

### Loss of TgCRCK1 does not attenuate the parasite’s virulence *in vivo*

**Fig 6:**
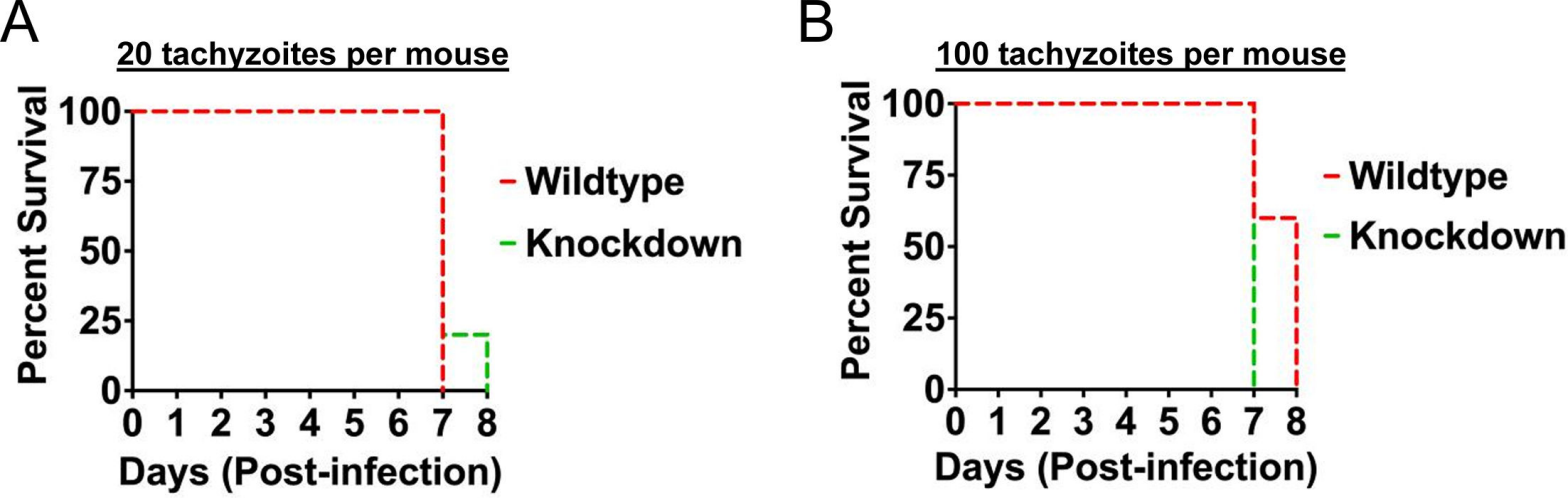
Loss of TgCRCK1 does not attenuate parasite virulence *in vivo*. Survival curves for female mice injected intraperitoneally with either (**A**) 20 or (**B**) 100 tachyzoites of TgCRCK1.mAID.HA strain. Five female BALB/c mice were included in each group. The animals were subsequently treated with either vehicle control or auxin orally and also intraperitoneally. ns, not significant. Log-rank (Mantel-Cox) test (GraphPad Prism).

Given the importance of TgCRCK1 for parasite fitness *in vitro*, we next sought to determine its role in *Toxoplasma* pathogenesis *in vivo*. To assess virulence, female BALB/c mice were inoculated either with 20 or 100 tachyzoites of TgCRCK1.mAID-HA strain per mouse via intra-peritoneal route (*n* = 5 mice/group). Auxin was administered orally and also through daily intraperitoneal injections to maintain continuous protein depletion in the knockdown groups (45). Strikingly, with both doses of parasite inoculum (including those treated with auxin or vehicle control), we observed animals succumbing to parasite infection in the first 8 days (Fig. 6A and B). These findings suggest that loss of TgCRCK1 does not attenuate *Toxoplasma* virulence in this animal model.

## DISCUSSION

*T. gondii* relies on a complex array of regulatory mechanisms to invade host cells, replicate, and propagate within its host (8, 9, 46, 47), and protein kinases play central roles in many of these events (16). In this study, we characterized TgCRCK1, a temporally regulated kinase, and demonstrated its importance in the *in vitro* lytic cycle, linking its contribution to cell division, host cell invasion, and gene regulation.

We found that TgCRCK1 localizes to the parasite cytoplasm and is exclusively expressed in the S and M/C phases of the cell cycle. Our findings that TgCRCK1 is a developmentally regulated cytoplasmic kinase are indeed in agreement with a previous study (20). Thus far, the precise mechanism involved in tight regulation of expression of cell cycle-regulated proteins in *Toxoplasma* has not been defined. However, since the protein expression pattern closely follows the transcriptomic expression profile (39), it is quite likely that the TgCRCK1 appearance in the parasite is controlled at the transcriptional level. Hence, investigating the promoter-proximal elements responsible for this regulation would be an interesting avenue for future research.

The genome-wide screen indicated that TgCRCK1 may be essential for *Toxoplasma* viability, as evidenced by a phenotype score of −4.25 (40). However, our plaque assays demonstrated that although TgCRCK1 plays a significant role in parasite growth, it is not indispensable for *Toxoplasma* fitness. This observation is not uncommon, as many proteins with negative phenotypic scores have been demonstrated to be non-essential for parasite propagation (43, 48, 49).

The timing of expression of TgCRCK1 is confined to the S and M/C phases during *Toxoplasma* cell cycle. This temporal regulation of expression of this kinase provided an initial inkling that TgCRCK1 could be playing a role in endodyogeny. Additionally, a small pool of this protein was found to be associated with the pellicle region of the newly forming daughter parasites. Accordingly, we did see that the loss of TgCRCK1 is associated with defects in endodyogeny that included vacuoles containing odd numbers of parasites as well as abnormal daughter cell buddings. To assess whether TgCRCK1 plays a role in egress, we examined the egress efficiency of TgCRCK1-depleted parasites using the calcium ionophore A23187. Although we did not observe a significant reduction in ionophore-induced egress, it remains possible that TgCRCK1 contributes to other egress pathways (50). Future studies using alternative egress stimuli or monitoring natural egress dynamics may help clarify whether TgCRCK1 is involved in this process.

In addition to defects in replication, we noticed some moderate defect in host-cell invasion capability of parasites lacking TgCRCK1. This invasion defect is likely an indirect consequence of TgCRCK1 deficiency, as the protein is not expressed during the G1 phase. The invasion defect seen during TgCRCK1 deficiency could be due to multiple reasons. Firstly, studies have shown that although a large percentage of the extracellular parasite population is in G1 phase of the cell cycle, there is a significant proportion of parasites in the S and M/C phases, as well (51). Hence, it is feasible that in those extracellular parasites in the S and M/C phases, lack of TgCRCK1 could be affecting parasite fitness leading to reduced invasion capacity. Second, it is also feasible that defects arising during endodyogeny due to absence of TgCRCK1 could have downstream effects that impair parasite invasion fitness.

Loss of TgCRCK1 also leads to dysregulation of gene expression in the parasite, although the number of affected genes and the magnitude of fold changes are relatively modest. Given that TgCRCK1 is a kinase, its primary role in parasite biology is likely mediated through phosphorylation of substrate proteins rather than direct transcriptional regulation. Therefore, the relatively subtle changes observed at the transcriptome level are not unexpected. To better understand the impact of TgCRCK1 on phosphorylation events, quantitative phosphoproteomics experiments will be essential (52). Combining phosphoproteomics data with techniques such as proximity labeling (53, 54) could help identify TgCRCK1 substrates and clarify the precise mechanisms by which this kinase contributes to *Toxoplasma* propagation.

Despite the modest transcriptomic effects, several downregulated genes are of particular interest. Notably, one of the most significantly downregulated genes encodes a kelch repeat-containing protein (TGGT1_229290). In the related apicomplexan *Plasmodium*, kelch domain proteins have been implicated in cytoskeletal organization, motility, and host cell invasion (55). Another downregulated gene, TGGT1_209970, encodes a member of the Spc97/Spc98 family, which is known to play a critical role in microtubule organization (56). Additionally, several genes involved in gene expression (TGGT1_254140, TGGT1_262750, TGGT1_203170, and TGGT1_258990) and metabolism (TGGT1_255200, TGGT1_231000, TGGT1_219630, and TGGT1_257480) are also downregulated. Many of the affected genes are annotated as hypothetical proteins, including TGGT1_220060A, TGGT1_225890, and TGGT1_254530, some of which are predicted to be essential for *Toxoplasma* fitness (40). Given the functional importance of these genes, it is not surprising that TgCRCK1 deficiency leads to a marked reduction in parasite propagation.

Although TgCRCK1 is critical for *Toxoplasma* growth *in vitro*, its loss did not affect parasite virulence in a murine infection model. This discrepancy could reflect incomplete TgCRCK1 knockdown *in vivo* following auxin treatment. However, because our findings are consistent with a CRISPR-based genome-wide screen that identified genes involved in acute toxoplasmosis in mice (57), this explanation is unlikely. A more plausible interpretation is that functional redundancy among parasite kinases may compensate for the absence of TgCRCK1. Alternatively, TgCRCK1 may primarily function during lytic cycle events, with limited involvement in host immune modulation. Nonetheless, these results do not exclude a potential role for TgCRCK1 in parasite pathogenesis. Notably, TgCRCK1 is also expressed in bradyzoites (58), suggesting it may contribute to chronic stages of infection. Future studies investigating its function in bradyzoite biology may uncover additional roles for this kinase in the *Toxoplasma* life cycle.

In summary, we have identified a cell cycle-regulated kinase, TgCRCK1, which is essential for *Toxoplasma* growth *in vitro*. Future studies aimed at elucidating the TgCRCK1 interactome and characterizing its candidate substrate proteins will provide deeper insights into the kinase’s mechanism of action and the signaling pathways it regulates.

## MATERIALS AND METHODS

### Host cells and parasite culture

*T. gondii* tachyzoites were maintained through continuous passaging in HFF cells within a humidified incubator set at 37°C with 5% CO_2_. The culture medium used was Dulbecco’s Modified Eagle’s Medium (DMEM) with 10% fetal bovine serum (FBS), L-glutamine (2 mM), and penicillin-streptomycin (50 µg/mL). Standard procedures were followed for parasite maintenance, harvesting, and purification (43, 44, 59).

### Endogenous tagging of TgCRCK1 and generation of TgCRCK1.mAID.HA strain

The endogenous tagging of TgCRCK1 at the C-terminus was performed according to previously published protocols (42, 60, 61). Briefly, a single guide RNA (sgRNA) plasmid containing a protospacer against the 3′ untranslated region (UTR) of TgCRCK1 downstream of the stop codon was generated through site-directed mutagenesis. The homology-directed repair (HDR) templates were PCR amplified using the vectors, p3XHA.LIC-DHFR and pmAID3xHA.LIC-HPT that contain HA and mAID.HA epitope tags, respectively. Additionally, both the plasmids contain a selection cassette that provides resistance against pyrimethamine (DHFR) and MPA-Xanthine (HXGPRT) (62, 63). The 60-bp primers used in generating the repair template include 40 bp of homology immediately upstream of the stop codon or 40 bp of homology within the 3′ UTR downstream of the CRISPR/Cas9 cut site. All primers that were used for pU6-Universal plasmids and HDR templates are listed in Table S1. The sgRNA plasmid and the HDR templates were then transfected into RHΔKu80 or RHΔKu80.TIR1 strain using nucleofector (35). Transfected parasites were then cultured in the presence of either pyrimethamine or MPA/xanthine to select stably transformed parasites that were cloned by limiting dilution. The clones were screened and validated by PCR and sequencing.

### Immunofluorescence microscopy

Immunofluorescence staining of the intracellular parasites was carried out as described previously (35, 64, 65). Primary antibodies used were mouse anti-HA (Cell Signaling Technology, Inc., 6E2, 1:250), rabbit anti-IMC3 (1:500) (35, 43), TgCentrin1 (1:250) (66) and TgISP1 (1:500) (67). Secondary antibodies were Alexa Fluor 594-conjugated goat anti-rabbit or Alexa Fluor 488-conjugated goat anti-mouse (35, 43) (Molecular Probes, 1:1,000). Imaging was performed using a Zeiss Axio Observer seven microscope (Carl Zeiss). Digital images were acquired utilizing Axiocam 506 mono charge-coupled device (CCD) camera using Axiovision software.

### Plaque assays

Plaque assays were performed as described previously with some modifications (44, 68). Intracellular parasites were harvested, syringe filtered, and added onto a confluent monolayer of HFF cells in a 12-well plate containing medium (DMEM with 10% FBS) with vehicle control or auxin (500 μM IAA). The plates were then incubated at 37°C for 6 days without any movement. The plates were then washed with PBS, methanol fixed, and stained with 2% crystal violet to visualize regions of host cell disruption. Plaques were imaged using the Molecular Imager Gel Doc XR system (Bio-Rad) and analyzed using Image Lab software. ImageJ was used to outline and quantify plaque areas. Experiments were conducted in triplicate to determine average plaque number and sizes.

### Replication assay

To assess the parasite doubling time, freshly egressed parasites were inoculated into confluent HFF monolayers in 12-well plates and allowed to invade for 2 h. The monolayers were then washed three times with medium (DMEM with 10% FBS) to remove uninvaded parasites and incubated at 37°C in DMEM with 10% FBS containing vehicle control or presence of 500 μM IAA. At 24 h post-infection, the cells were fixed with methanol and stained using Diff-Quik (Dade-Behring) according to the manufacturer’s instructions. For each treatment, at least 100 vacuoles from three biological replicates were assessed for the number of parasites per vacuole.

### Ionophore-induced egress assay

Egress efficiency following calcium ionophore treatment was assessed following established protocols (44, 69, 70). Freshly harvested parasites were added to confluent HFF monolayers in 24-well plates at a multiplicity of infection (MOI) of 1 and then incubated at 37°C for 28 h. The cultures were then treated with vehicle control or auxin for 4 h, and egress was induced using calcium ionophore A23187 (1 µM) in Hanks’ Balanced Salt Solution (HBSS) at 37°C for 2 minutes. Cultures were then fixed with methanol and stained using Diff-Quik (Dade-Behring). Egress percentage was calculated by dividing the number of lysed vacuoles by the total vacuole count per sample.

### Invasion assays

Invasion assays were performed in eight-well chamber slides as described previously with the following modifications (71, 72). Briefly, parasites were pretreated with vehicle control or 500 μM IAA (to deplete the mAID-3HA tagged TgCRCK1) in DMEM with 10%FBS for 4 h. Purified tachyzoites were then added onto HFF monolayers (2 × 10^6^ parasites well) and incubated at 37°C for 30 min in the presence or absence of auxin to allow invasion. Slides were then washed three times to remove non-invaded parasites, fixed, blocked, and stained with mouse anti-SAG1 without permeabilization. After 1 h, slides were washed, permeabilized with 0.01% TX-100 and stained with rabbit anti-M2AP antibody. The slides were further washed and stained with secondary antibodies, Alexa Fluor-594-conjugated goat anti-mouse (Molecular Probes) and Alexa Fluor-488-conjugated goat anti-rabbit (Molecular Probes). After 1 h, slides were washed and mounted using Vectashield (with DAPI). Parasites that were both red and green were identified as extracellular (attached), whereas those that were green but not red were identified as intracellular (invaded). Images of 10 random fields of view within each well were captured at 600× magnification, and the total number of intracellular parasites and host cell nuclei were enumerated.

### RNA sequencing and differential gene expression analysis

RNA sequencing was performed according to previously published protocols with some modifications (35, 73). Total RNA from intracellular TgCRCK1.mAID.HA parasites treated with 500 μM IAA or vehicle control for 4 h was isolated using the RNeasy kit (Qiagen). RNA samples were obtained from three independent experiments. The quality of total RNA samples was verified by using a BioAnalyzer (Agilent), followed by digestion with DNAse I (NEB). Ribosomal RNA was removed using the Ribo-Zero rRNA removal kit (human/mouse/rat, Illumina). Sequencing libraries were then generated using the TruSeq RNA Sample Prep Kit (v2, Illumina) according to manufacturer’s protocol. Libraries were amplified using the TruSeq Cluster Kit (v3, Illumina), and subjected to 50-bp single-end sequencing with the Illumina HiSeq 2000 system. Sequencing reads were aligned to the *Toxoplasma* GT1 reference genome (ToxoDB v.53, https://toxodb.org/toxo/app) using the STAR software package (v.2.7.1a , with default settings) (74). Filtered and normalized gene expression levels were calculated from the aligned reads using HTSeq v.0.13.5 (75). Differentially expressed genes were identified by linear modeling and Bayesian statistics using the limma package for R v.3.49.1, (76).

### *In vivo* virulence assay

The virulence assays were performed according to previously published studies (77). All laboratory animal work in this study was conducted in compliance with guidelines from the Virginia Tech Committee on the Use and Care of Animals (IACUC) (protocol no. 23-163). Female 6-week-old BALB/c mice (Jackson Laboratories) were intraperitoneally injected with TgCRCK1.mAID-HA parasites (20 or 100 tachyzoites per mouse). For the knockdown groups, auxin was provided in the drinking water (500 mg/L) and given intraperitoneally (150 mg/kg) every day throughout the experiment (77). Parasite viability was confirmed immediately post-infection through plaque assays from the same preparation used for mouse injections. Mice were monitored throughout the experiment for disease symptoms, and survival was recorded.

## SUPPLEMENTAL MATERIAL

Figure S1 spectrum.02691-25-s0001.tiff IFA analysis of TgCRCK1.mAID.HA strain in presence or absence of auxin using antibodies against HA epitope and different organellar proteins.

Supplemental legends spectrum.02691-25-s0002.docx Legends for Figure S1, and Tables S1 and S2.

Table S1 spectrum.02691-25-s0003.pdf Primers used in this study.

Table S2 spectrum.02691-25-s0004.xlsx Genes dysregulated in the TgCRCK1 knockdown mutant.

ASM does not own the copyrights to Supplemental Material that may be linked to, or accessed through, an article. The authors have granted ASM a non-exclusive, world-wide license to publish the Supplemental Material files. Please contact the corresponding author directly for reuse.

## References

[1] Weiss LM, Dubey JP. 2009. Toxoplasmosis: a history of clinical observations. Int J Parasitol 39:895–901. doi:10.1016/j.ijpara.2009.02.004 19217908 PMC2704023

[2] Halonen SK, Weiss LM. 2013. Toxoplasmosis. Handb Clin Neurol 114:125–145. doi:10.1016/B978-0-444-53490-3.00008-X 23829904 PMC4157368

[3] Dubey JP. 2002. A review of toxoplasmosis in wild birds. Vet Parasitol (Amst) 106:121–153. doi:10.1016/S0304-4017(02)00034-1 12031816

[4] Dubey JP. 1998. Advances in the life cycle of Toxoplasma gondii. Int J Parasitol 28:1019–1024. doi:10.1016/s0020-7519(98)00023-x 9724872

[5] Smith JL. 1997. Long-term consequences of foodborne toxoplasmosis: effects on the unborn, the immunocompromised, the elderly, and the immunocompetent. J Food Prot 60:1595–1611. doi:10.4315/0362-028X-60.12.1595 31207758

[6] Luft BJ, Remington JS. 1992. Toxoplasmic encephalitis in AIDS. Clin Infect Dis 15:211–222. doi:10.1093/clinids/15.2.211 1520757

[7] Jones JL, Lopez A, Wilson M, Schulkin J, Gibbs R. 2001. Congenital toxoplasmosis: a review. Obstet Gynecol Surv 56:296–305. doi:10.1097/00006254-200105000-00025 11333376

[8] Blader IJ, Coleman BI, Chen C-T, Gubbels M-J. 2015. Lytic Cycle of Toxoplasma gondii: 15 years later. Annu Rev Microbiol 69:463–485. doi:10.1146/annurev-micro-091014-104100 26332089 PMC4659696

[9] Black MW, Boothroyd JC. 2000. Lytic cycle of Toxoplasma gondii. Microbiol Mol Biol Rev 64:607–623. doi:10.1128/MMBR.64.3.607-623.2000 10974128 PMC99006

[10] Gubbels M-J, Coppens I, Zarringhalam K, Duraisingh MT, Engelberg K. 2021. The modular circuitry of apicomplexan cell division plasticity. Front Cell Infect Microbiol 11:670049. doi:10.3389/fcimb.2021.670049 33912479 PMC8072463

[11] Striepen B, Jordan CN, Reiff S, van Dooren GG. 2007. Building the perfect parasite: cell division in apicomplexa. PLoS Pathog 3:e78. doi:10.1371/journal.ppat.0030078 17604449 PMC1904476

[12] Hortua Triana MA, Márquez-Nogueras KM, Vella SA, Moreno SNJ. 2018. Calcium signaling and the lytic cycle of the apicomplexan parasite Toxoplasma gondii. Biochim Biophys Acta Mol Cell Res 1865:1846–1856. doi:10.1016/j.bbamcr.2018.08.004 30992126 PMC6477927

[13] Mendez OA, Koshy AA. 2017. Toxoplasma gondii: entry, association, and physiological influence on the central nervous system. PLoS Pathog 13:e1006351. doi:10.1371/journal.ppat.1006351 28727854 PMC5519211

[14] Piro F, Focaia R, Dou Z, Masci S, Smith D, Di Cristina M. 2021. An uninvited seat at the dinner table: how apicomplexan parasites scavenge nutrients from the host. Microorganisms 9:2592. doi:10.3390/microorganisms9122592 34946193 PMC8707601

[15] Peixoto L, Chen F, Harb OS, Davis PH, Beiting DP, Brownback CS, Ouloguem D, Roos DS. 2010. Integrative genomic approaches highlight a family of parasite-specific kinases that regulate host responses. Cell Host & Microbe 8:208–218. doi:10.1016/j.chom.2010.07.004 20709297 PMC2963626

[16] Gaji RY, Sharp AK, Brown AM. 2021. Protein kinases in Toxoplasma gondii. Int J Parasitol 51:415–429. doi:10.1016/j.ijpara.2020.11.006 33581139 PMC11065138

[17] Behnke MS, Dubey JP, Sibley LD. 2016. Genetic mapping of pathogenesis determinants in Toxoplasma gondii. Annu Rev Microbiol 70:63–81. doi:10.1146/annurev-micro-091014-104353 27359216

[18] Chen C-T, Gubbels M-J. 2013. The Toxoplasma gondii centrosome is the platform for internal daughter budding as revealed by a Nek1 kinase mutant. J Cell Sci 126:3344–3355. doi:10.1242/jcs.123364 23729737 PMC3730244

[19] O’Shaughnessy WJ, Hu X, Henriquez SA, Reese ML. 2023. Toxoplasma ERK7 protects the apical complex from premature degradation. J Cell Biol 222:e202209098. doi:10.1083/jcb.202209098 37027006 PMC10083718

[20] Smith TA, Lopez-Perez GS, Herneisen AL, Shortt E, Lourido S. 2022. Screening the Toxoplasma kinome with high-throughput tagging identifies a regulator of invasion and egress. Nat Microbiol 7:868–881. doi:10.1038/s41564-022-01104-0 35484233 PMC9167752

[21] Ojo KK, Vinayak S. 2025. Molecular and biochemical characterization of parasites protein phosphorylation: emerging trends, challenges and opportunities. Mol Biochem Parasitol 262:111675. doi:10.1016/j.molbiopara.2025.111675 39884464

[22] Berry L, Chen C-T, Reininger L, Carvalho TG, El Hajj H, Morlon-Guyot J, Bordat Y, Lebrun M, Gubbels M-J, Doerig C, Daher W. 2016. The conserved apicomplexan aurora kinase TgArk3 is involved in endodyogeny, duplication rate and parasite virulence. Cell Microbiol 18:1106–1120. doi:10.1111/cmi.12571 26833682 PMC4961599

[23] Saeij JPJ, Boyle JP, Coller S, Taylor S, Sibley LD, Brooke-Powell ET, Ajioka JW, Boothroyd JC. 2006. Polymorphic secreted kinases are key virulence factors in toxoplasmosis. Science 314:1780–1783. doi:10.1126/science.1133690 17170306 PMC2646183

[24] Beraki T, Hu X, Broncel M, Young JC, O’Shaughnessy WJ, Borek D, Treeck M, Reese ML. 2019. Divergent kinase regulates membrane ultrastructure of the Toxoplasma parasitophorous vacuole. Proc Natl Acad Sci USA 116:6361–6370. doi:10.1073/pnas.1816161116 30850550 PMC6442604

[25] Hawkins LM, Wang C, Chaput D, Batra M, Marsilia C, Awshah D, Suvorova ES. 2024. The Crk4-Cyc4 complex regulates G_2_/M transition in Toxoplasma gondii. EMBO J 43:2094–2126. doi:10.1038/s44318-024-00095-4 38600241 PMC11148040

[26] Skariah S, Walwyn O, Engelberg K, Gubbels M-J, Gaylets C, Kim N, Lynch B, Sultan A, Mordue DG. 2016. The FIKK kinase of Toxoplasma gondii is not essential for the parasite’s lytic cycle. Int J Parasitol 46:323–332. doi:10.1016/j.ijpara.2016.01.001 26859096 PMC4844859

[27] Lourido S, Tang K, Sibley LD. 2012. Distinct signalling pathways control Toxoplasma egress and host-cell invasion. EMBO J 31:4524–4534. doi:10.1038/emboj.2012.299 23149386 PMC3545288

[28] McCoy JM, Whitehead L, van Dooren GG, Tonkin CJ. 2012. TgCDPK3 regulates calcium-dependent egress of Toxoplasma gondii from host cells. PLoS Pathog 8:e1003066. doi:10.1371/journal.ppat.1003066 23226109 PMC3514314

[29] Garrison E, Treeck M, Ehret E, Butz H, Garbuz T, Oswald BP, Settles M, Boothroyd J, Arrizabalaga G. 2012. A forward genetic screen reveals that calcium-dependent protein kinase 3 regulates egress in Toxoplasma. PLoS Pathog 8:e1003049. doi:10.1371/journal.ppat.1003049 23209419 PMC3510250

[30] White MW, Suvorova ES. 2018. Apicomplexa cell cycles: something old, borrowed, lost, and new. Trends Parasitol 34:759–771. doi:10.1016/j.pt.2018.07.006 30078701 PMC6157590

[31] Brown KM, Suvorova E, Farrell A, McLain A, Dittmar A, Wiley GB, Marth G, Gaffney PM, Gubbels MJ, White M, Blader IJ. 2014. Forward genetic screening identifies a small molecule that blocks Toxoplasma gondii growth by inhibiting both host- and parasite-encoded kinases. PLoS Pathog 10:e1004180. doi:10.1371/journal.ppat.1004180 24945800 PMC4055737

[32] Suvorova ES, Francia M, Striepen B, White MW. 2015. A novel bipartite centrosome coordinates the apicomplexan cell cycle. PLoS Biol 13:e1002093. doi:10.1371/journal.pbio.1002093 25734885 PMC4348508

[33] Berry L, Chen C-T, Francia ME, Guerin A, Graindorge A, Saliou J-M, Grandmougin M, Wein S, Bechara C, Morlon-Guyot J, Bordat Y, Gubbels M-J, Lebrun M, Dubremetz J-F, Daher W. 2018. Toxoplasma gondii chromosomal passenger complex is essential for the organization of a functional mitotic spindle: a prerequisite for productive endodyogeny. Cell Mol Life Sci 75:4417–4443. doi:10.1007/s00018-018-2889-6 30051161 PMC6260807

[34] O’Shaughnessy WJ, Hu X, Beraki T, McDougal M, Reese ML. 2020. Loss of a conserved MAPK causes catastrophic failure in assembly of a specialized cilium-like structure in Toxoplasma gondii Mol Biol Cell 31:881–888. doi:10.1091/mbc.E19-11-0607 32073987 PMC7185968

[35] Montano H, Anandkrishnan R, Carruthers VB, Gaji RY. 2023. TgTKL4 is a novel kinase that plays an important role in Toxoplasma morphology and fitness. mSphere 8:e0064922. doi:10.1128/msphere.00649-22 36786615 PMC10117109

[36] Hu X, O’Shaughnessy WJ, Beraki TG, Reese ML. 2020. Loss of the conserved alveolate kinase MAPK2 decouples Toxoplasma cell growth from cell division. mBio 11:e02517-20. doi:10.1128/mBio.02517-20 33173004 PMC7667025

[37] Karki S, Barth ZK, Aylward FO. 2025. Ancient host-virus gene transfer hints at a diverse pre-LECA virosphere. J Mol Evol 93:295–305. doi:10.1007/s00239-025-10246-8 40298963 PMC12198294

[38] Levine ND. 1977. Tazonomy of Toxoplasma. J Protozool 24:36–41. doi:10.1111/j.1550-7408.1977.tb05278.x 325203

[39] Behnke MS, Wootton JC, Lehmann MM, Radke JB, Lucas O, Nawas J, Sibley LD, White MW. 2010. Coordinated progression through two subtranscriptomes underlies the tachyzoite cycle of Toxoplasma gondii. PLoS One 5:e12354. doi:10.1371/journal.pone.0012354 20865045 PMC2928733

[40] Sidik SM, Huet D, Ganesan SM, Huynh M-H, Wang T, Nasamu AS, Thiru P, Saeij JPJ, Carruthers VB, Niles JC, Lourido S. 2016. A genome-wide CRISPR screen in Toxoplasma identifies essential apicomplexan genes. Cell 166:1423–1435. doi:10.1016/j.cell.2016.08.019 27594426 PMC5017925

[41] Huynh M-H, Carruthers VB. 2009. Tagging of endogenous genes in a Toxoplasma gondii strain lacking Ku80. Eukaryot Cell 8:530–539. doi:10.1128/EC.00358-08 19218426 PMC2669203

[42] Brown KM, Long S, Sibley LD. 2018. Conditional knockdown of proteins using auxin-inducible degron (AID) fusions in Toxoplasma gondii. Bio Protoc 8:4. doi:10.21769/BioProtoc.2728 PMC5890294 29644255

[43] Varberg JM, Coppens I, Arrizabalaga G, Gaji RY. 2018. TgTKL1 is a unique plant-like nuclear kinase that plays an essential role in acute toxoplasmosis. mBio 9:e00301-18. doi:10.1128/mBio.00301-18 29559568 PMC5874906

[44] Ali DH, Anandakrishnan R, Carruthers VB, Gaji RY. 2024. Kinase function of TgTKL1 is essential for its role in Toxoplasma propagation and pathogenesis. mSphere 9:e0077924. doi:10.1128/msphere.00779-24 39475314 PMC11580469

[45] Wan W, Dong H, Lai D-H, Yang J, He K, Tang X, Liu Q, Hide G, Zhu X-Q, Sibley LD, Lun Z-R, Long S. 2023. The Toxoplasma micropore mediates endocytosis for selective nutrient salvage from host cell compartments. Nat Commun 14:977. doi:10.1038/s41467-023-36571-4 36813769 PMC9947163

[46] Sibley DL, Charron A, Håkansson S, Mordue D. 2013. Invasion and intracellular survival by *Toxoplasma*. In Madame Curie Bioscience Database [Internet]. Landes Bioscience.

[47] Matta SK, Rinkenberger N, Dunay IR, Sibley LD. 2021. Toxoplasma gondii infection and its implications within the central nervous system. Nat Rev Microbiol 19:467–480. doi:10.1038/s41579-021-00518-7 33627834

[48] Moss WJ, Patterson CE, Jochmans AK, Brown KM. 2022. Functional analysis of the expanded phosphodiesterase gene family in Toxoplasma gondii tachyzoites. mSphere 7:e0079321. doi:10.1128/msphere.00793-21 35107337 PMC8809380

[49] Padilla LFA, Murray JM, Hu K. 2024. The initiation and early development of the tubulin-containing cytoskeleton in the human parasite Toxoplasma gondii Mol Biol Cell 35:ar37. doi:10.1091/mbc.E23-11-0418 38170577 PMC10916856

[50] Vella SA, Moore CA, Li Z-H, Hortua Triana MA, Potapenko E, Moreno SNJ. 2021. The role of potassium and host calcium signaling in Toxoplasma gondii egress. Cell Calcium 94:102337. doi:10.1016/j.ceca.2020.102337 33524795 PMC7914212

[51] Batra M, Marsilia C, Awshah D, Hawkins LM, Wang C, Chaput D, Naumova DA, Suvorova ES. 2025. Deciphering cell cycle organization of Toxoplasma endodyogeny. mBio 16:e0111925. doi:10.1128/mbio.01119-25 40590555 PMC12345243

[52] Treeck M, Sanders JL, Gaji RY, LaFavers KA, Child MA, Arrizabalaga G, Elias JE, Boothroyd JC. 2014. The calcium-dependent protein kinase 3 of Toxoplasma influences basal calcium levels and functions beyond egress as revealed by quantitative phosphoproteome analysis. PLoS Pathog 10:e1004197. doi:10.1371/journal.ppat.1004197 24945436 PMC4063958

[53] Chen AL, Kim EW, Toh JY, Vashisht AA, Rashoff AQ, Van C, Huang AS, Moon AS, Bell HN, Bentolila LA, Wohlschlegel JA, Bradley PJ. 2015. Novel components of the Toxoplasma inner membrane complex revealed by BioID. mBio 6:e02357–14. doi:10.1128/mBio.02357-14 25691595 PMC4337574

[54] Gaji RY, Johnson DE, Treeck M, Wang M, Hudmon A, Arrizabalaga G. 2015. Phosphorylation of a myosin motor by TgCDPK3 facilitates rapid initiation of motility during Toxoplasma gondii egress. PLoS Pathog 11:e1005268. doi:10.1371/journal.ppat.1005268 26544049 PMC4636360

[55] Guttery DS, Poulin B, Ferguson DJP, Szöőr B, Wickstead B, Carroll PL, Ramakrishnan C, Brady D, Patzewitz E-M, Straschil U, Solyakov L, Green JL, Sinden RE, Tobin AB, Holder AA, Tewari R. 2012. A unique protein phosphatase with kelch-like domains (PPKL) in Plasmodium modulates ookinete differentiation, motility and invasion. PLoS Pathog 8:e1002948. doi:10.1371/journal.ppat.1002948 23028336 PMC3447748

[56] Knop M, Pereira G, Geissler S, Grein K, Schiebel E. 1997. The spindle pole body component Spc97p interacts with the gamma-tubulin of Saccharomyces cerevisiae and functions in microtubule organization and spindle pole body duplication. EMBO J 16:1550–1564. doi:10.1093/emboj/16.7.1550 9130700 PMC1169759

[57] Giuliano CJ, Wei KJ, Harling FM, Waldman BS, Farringer MA, Boydston EA, Lan TCT, Thomas RW, Herneisen AL, Sanderlin AG, Coppens I, Dvorin JD, Lourido S. 2024. CRISPR-based functional profiling of the Toxoplasma gondii genome during acute murine infection. Nat Microbiol 9:2323–2343. doi:10.1038/s41564-024-01754-2 38977907 PMC11811839

[58] Pittman KJ, Aliota MT, Knoll LJ. 2014. Dual transcriptional profiling of mice and Toxoplasma gondii during acute and chronic infection. BMC Genomics 15:806. doi:10.1186/1471-2164-15-806 25240600 PMC4177681

[59] Gaji RY, Behnke MS, Lehmann MM, White MW, Carruthers VB. 2011. Cell cycle-dependent, intercellular transmission of Toxoplasma gondii is accompanied by marked changes in parasite gene expression. Mol Microbiol 79:192–204. doi:10.1111/j.1365-2958.2010.07441.x 21166903 PMC3075969

[60] Shen B, Brown K, Long S, Sibley LD. 2017. Development of CRISPR/Cas9 for efficient genome editing in Toxoplasma gondii. Methods Mol Biol 1498:79–103. doi:10.1007/978-1-4939-6472-7_6 27709570

[61] Sidik SM, Hackett CG, Tran F, Westwood NJ, Lourido S. 2014. Efficient genome engineering of Toxoplasma gondii using CRISPR/Cas9. PLoS One 9:e100450. doi:10.1371/journal.pone.0100450 24971596 PMC4074098

[62] Donald RGK, Roos DS. 1993. Stable molecular transformation of Toxoplasma gondii: a selectable dihydrofolate reductase-thymidylate synthase marker based on drug-resistance mutations in malaria. Proc Natl Acad Sci USA 90:11703–11707. doi:10.1073/pnas.90.24.11703 8265612 PMC48052

[63] Knoll LJ, Boothroyd JC. 1998. Isolation of developmentally regulated genes from Toxoplasma gondii by a gene trap with the positive and negative selectable marker hypoxanthine-xanthine-guanine phosphoribosyltransferase. Mol Cell Biol 18:807–814. doi:10.1128/MCB.18.2.807 9447977 PMC108792

[64] Gaji RY, Flammer HP, Carruthers VB. 2011. Forward targeting of Toxoplasma gondii proproteins to the micronemes involves conserved aliphatic amino acids. Traffic 12:840–853. doi:10.1111/j.1600-0854.2011.01192.x 21438967 PMC3115430

[65] Carruthers VB, Sibley LD. 1999. Mobilization of intracellular calcium stimulates microneme discharge in Toxoplasma gondii. Mol Microbiol 31:421–428. doi:10.1046/j.1365-2958.1999.01174.x 10027960

[66] Chen C-T, Gubbels M-J. 2019. TgCep250 is dynamically processed through the division cycle and is essential for structural integrity of the Toxoplasma centrosome. Mol Biol Cell 30:1160–1169. doi:10.1091/mbc.E18-10-0608 30865554 PMC6724518

[67] Beck JR, Rodriguez-Fernandez IA, Cruz de Leon J, Huynh M-H, Carruthers VB, Morrissette NS, Bradley PJ. 2010. A novel family of Toxoplasma IMC proteins displays a hierarchical organization and functions in coordinating parasite division. PLoS Pathog 6:e1001094. doi:10.1371/journal.ppat.1001094 20844581 PMC2936552

[68] Mazumdar J, H Wilson E, Masek K, A Hunter C, Striepen B. 2006. Apicoplast fatty acid synthesis is essential for organelle biogenesis and parasite survival in Toxoplasma gondii. Proc Natl Acad Sci USA 103:13192–13197. doi:10.1073/pnas.0603391103 16920791 PMC1559775

[69] Gaji RY, Checkley L, Reese ML, Ferdig MT, Arrizabalaga G. 2014. Expression of the essential kinase PfCDPK1 from Plasmodium falciparum in Toxoplasma gondii facilitates the discovery of novel antimalarial drugs. Antimicrob Agents Chemother 58:2598–2607. doi:10.1128/AAC.02261-13 24550330 PMC3993251

[70] Stommel EW, Ely KH, Schwartzman JD, Kasper LH. 1997. Toxoplasma gondii:dithiol-induced Ca2+flux causes egress of parasites from the parasitophorous vacuole. Exp Parasitol 87:88–97. doi:10.1006/expr.1997.4187 9326884

[71] Huynh M-H. 2003. Rapid invasion of host cells by Toxoplasma requires secretion of the MIC2-M2AP adhesive protein complex. Embo J 22:2082–2090. doi:10.1093/emboj/cdg217 12727875 PMC156089

[72] Thornton LB, Key M, Micchelli C, Stasic AJ, Kwain S, Floyd K, Moreno SNJ, Dominy BN, Whitehead DC, Dou Z. 2023. A cathepsin C-like protease mediates the post-translation modification of Toxoplasma gondii secretory proteins for optimal invasion and egress. mBio 14:e0017423. doi:10.1128/mbio.00174-23 37326431 PMC10470614

[73] Varberg JM, Coppens I, Arrizabalaga G, Gaji RY. 2018. TgTKL1 is a unique plant-like nuclear kinase that plays an essential role in acute toxoplasmosis. mBio 9. doi:10.1128/mBio.00301-18 PMC5874906 29559568

[74] Dobin A, Davis CA, Schlesinger F, Drenkow J, Zaleski C, Jha S, Batut P, Chaisson M, Gingeras TR. 2013. STAR: ultrafast universal RNA-seq aligner. Bioinformatics 29:15–21. doi:10.1093/bioinformatics/bts635 23104886 PMC3530905

[75] Anders S, Pyl PT, Huber W. 2015. HTSeq—a python framework to work with high-throughput sequencing data. Bioinformatics 31:166–169. doi:10.1093/bioinformatics/btu638 25260700 PMC4287950

[76] Ritchie ME, Phipson B, Wu D, Hu Y, Law CW, Shi W, Smyth GK. 2015. Limma powers differential expression analyses for RNA-sequencing and microarray studies. Nucleic Acids Res 43:e47–e47. doi:10.1093/nar/gkv007 25605792 PMC4402510

[77] Brown KM, Sibley LD. 2018. Essential cGMP signaling in Toxoplasma is initiated by a hybrid P-type ATPase-Guanylate Cyclase. Cell Host Microbe 24:804–816. doi:10.1016/j.chom.2018.10.015 30449726 PMC6292738

