# Supplementary material for: The cell cycle-regulated cytoplasmic kinase, TgCRCK1, is required for efficient propagation of human protozoan pathogen, Toxoplasma gondii
Source: Microbiol Spectr. 2025 Dec 31;14(2):e02691-25. doi: 10.1128/spectrum.02691-25 (PMC12889055; doi:10.1128/spectrum.02691-25)
Supplement: Supplemental legends — Legends for Figure S1, and Tables S1 and S2. [file spectrum.02691-25-s0002.docx]

**Legends for supplemental material**

**LEGENDS FOR SUPPLEMENTAL FIGURES**

**Figure S1**. IFA analysis of TgCRCK1.mAID.HA strain in presence or absence of auxin using antibodies against HA epitope and different organellar proteins. The different markers tested include: **(A)** Centrin 1 as the marker for the centrosome, **(B)** ISP1 as a marker for apical cap. The top two panels are intracellular parasites treated with vehicle control and the bottom two panels are intracellular parasites treated with auxin. Scale bar, 2 μm.

**SUPPLEMENTAL DATA LEGENDS**

**1.** **Supplemental table S1.** List of primers used in this study.

**2. Supplemental table S2.** List of genes dysregulated in TgCRCK1 conditional knockdown mutant strain.
