# Supplementary material for: The cell cycle-regulated cytoplasmic kinase, TgCRCK1, is required for efficient propagation of human protozoan pathogen, Toxoplasma gondii
Source: Microbiol Spectr. 2025 Dec 31;14(2):e02691-25. doi: 10.1128/spectrum.02691-25 (PMC12889055; doi:10.1128/spectrum.02691-25)
Supplement: Table S1 — Primers used in this study. [file spectrum.02691-25-s0003.pdf]

Table S1. Primers used in this study.

S. No	Name	Sequence
1	CRCK1.sgRNA.F	tacagtatctGTTTTAGAGCTAGAAATAGC
2	CRCK1.sgRNA.R	catgctgatcAACTTGACATCCCCATTTAC
3	CRCK1.HA.RT.F	CATGTCGACACAGCAGCCCCCGGAAGCCGTGGAAGGGGTCggaagtggaggacgggaatt
4	CRCK1.HA.RT.R	GCTACACTGCACCATTTACCGGTTCCTTCACACACTCCCGACGGCCAGTGAATTGTAATA
5	CRCK1.mAID.RT.F	CATGTCGACACAGCAGCCCCCGGAAGCCGTGGAAGGGGTCatggtgagcaagggcgagga
6	CRCK1.mAID.RT.R	GCTACACTGCACCATTTACCGGTTCCTTCACACACTCCcgctctagactgcagataac
